# Supplementary material for: Entropy engineering promotes thermoelectric performance in p-type chalcogenides
Source: Nat Commun. 2021 May 28;12:3234. doi: 10.1038/s41467-021-23569-z (PMC8163856; doi:10.1038/s41467-021-23569-z)
Supplement: Supplementary file 1 — Supplementary Information [file 41467_2021_23569_MOESM1_ESM.pdf]

Supplementary Information

Entropy engineering promotes thermoelectric performance in p-type chalcogenides

Binbin Jiang,[1,5] Yong Yu,[1,5] Hongyi Chen,[2] Juan Cui,[1] Xixi Liu,[1] Lin Xie,[1] and Jiaqing He[1,3,4*]

[1]Shenzhen Key Laboratory of Thermoelectric Materials, Department of Physics, Southern University of Science and Technology, Shenzhen, 518055, China.
[2]College of Chemistry and Chemical Engineering, Central South University, Changsha, 410083, China.
[3]Guangdong-Hong Kong-Macao Joint Laboratory for Photonic-Thermal-Electrical Energy Materials and Devices, Southern University of Science and Technology, Shenzhen 518055, China
[4]Key Laboratory of Energy Conversion and Storage Technologies, Southern University of Science and Technology, Ministry of Education, Shenzhen, 518055, China.
[5]These authors contributed equally: B. Jiang, Y. Yu.

[*]Correspondence to：hejq@sustech.edu.cn **(J.Q.H.)**

Supplementary Methods

Calculation details

Density functional theory (DFT) calculations were performed within the Vienna ab initio simulation package (VASP)[1], using the projector augmented wave (PAW) method, the Perdew-Burke-Ernzerhof (PBE) functional and the generalized gradient approximation (GGA)[2]. We employed an energy cutoff of 400eV and a k-point density of $2\pi\times0.027$ Å^{-1} using the Γ-centered Monkhorst-Pack scheme[3]. A 3×3×3 supercell containing 54 atoms was constructed for band structure calculation. The special quasi-random structures (SQS) of high entropy alloys were generated using the mcsqs tool as implemented in the alloy theoretic automated toolkit (ATAT)[4]. The atoms and the structures were fully relaxed until the total energy converges within 10^{-5} eV and the force converges in 0.01 eV/Å. The spin-orbit coupling (SOC) effect was included in our calculations.

A 2×2×2 supercell was calculated for PbSe/CdS deformation energy, and 2×2×6 supercell was calculated for PbSe/CdS surface energy. The Brillouin zone integration was carried out on Monkhorst–Pack k-point meshes[3] with 3×3×3, 5×5×1, k-points for deformation energy and surface energy respectively. The plane-wave cutoff energy is 500 eV and the energy convergence criterion is 1×10^{-4} eV for the local density approximation calculations[5].

STEM/TEM characterization

The samples for STEM/TEM characterization were prepared by the standard mechanical grinding, polishing down to a thickness about 40 μm, and followed by ion milling at -170 ℃. During ion milling, the samples were milled with the beam voltage 4.2 kV and milling angle ±5°. After the sample was perforated, a voltage lower than 1 kV was used to optimize the thin area of the specimen. The samples were further milled lower than 0.5 kV to remove the surface damaged layer. STEM experiments were conducted on a Thermo Fisher Themis G260-300 electron microscope under 300 kV. The convergence angle and collection angle for the STEM HAADF imaging are 25 and 70–200 mrad, respectively. Strain analysis of the STEM and HAADF images were carried out by using the commercial Geometric Phase

Analysis package by HREM Research.

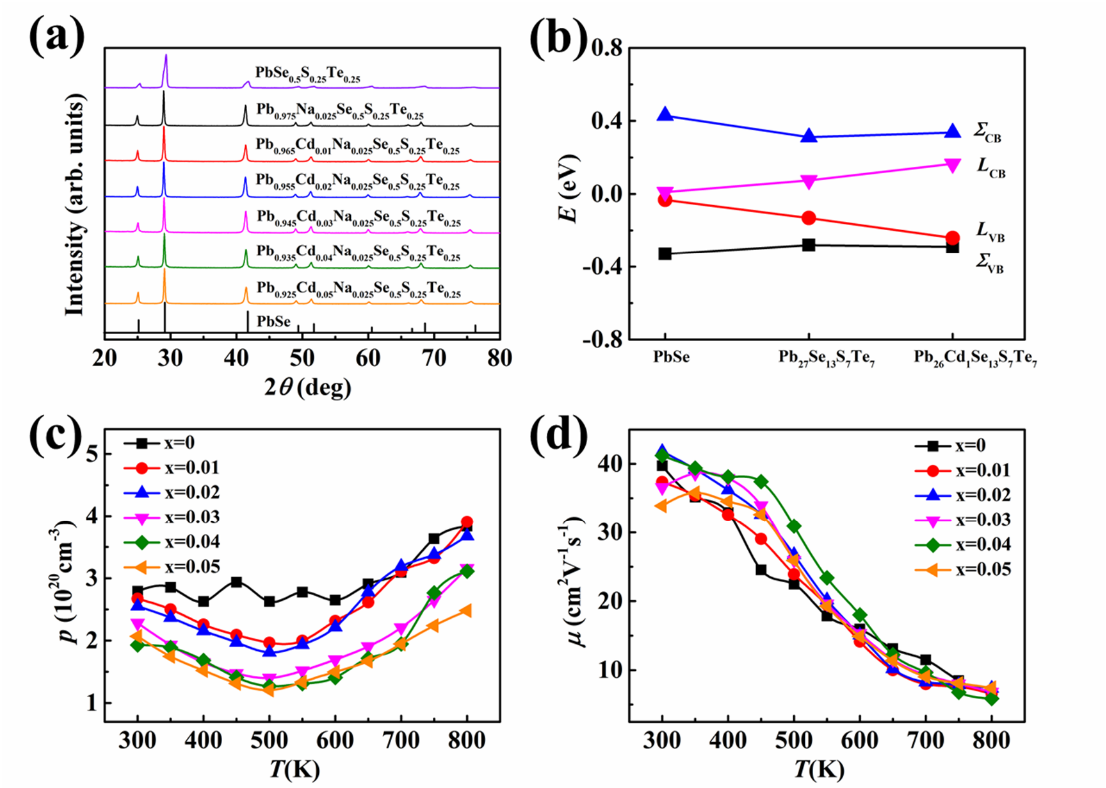

Supplementary Figure 1. (a) Powder XRD patterns in 20-80 degree range for $PbSe_{0.5}S_{0.25}Te_{0.25}$ and $Pb_{0.975-x}Cd_xNa_{0.025}Se_{0.5}S_{0.25}Te_{0.25}$ (x = 0, 0.01, 0.02, 0.03, 0.04, 0.05) samples. (b) Band energy difference of different conduction and valence bands for different composition. Temperature dependence of (c) hole concentration, and (d) hole mobility for $Pb_{0.975-x}Cd_xNa_{0.025}Se_{0.5}S_{0.25}Te_{0.25}$ (x = 0, 0.01, 0.02, 0.03, 0.04, 0.05) samples.

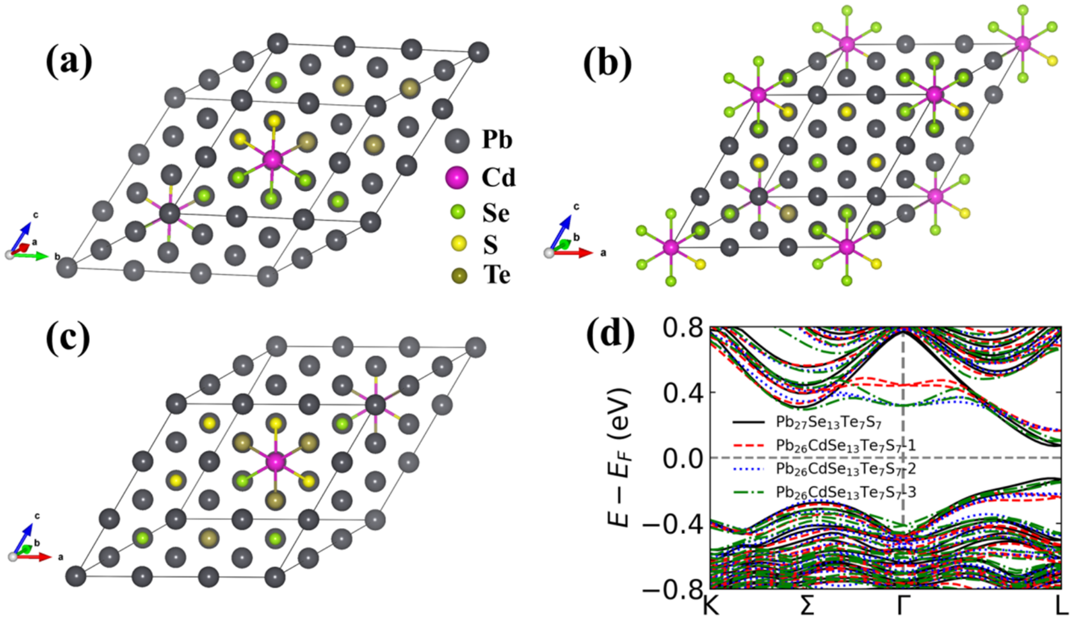

Supplementary Figure 2. (a)-(c): $Pb_{26}CdSe_{13}Te_7S_7$ with Cd at different positions, marked as $Pb_{26}CdSe_{13}Te_7S_7$-1, $Pb_{26}CdSe_{13}Te_7S_7$-2 and $Pb_{26}CdSe_{13}Te_7S_7$-3, respectively. Cd atom is surrounded by (a) 3 Se atoms, 2 S atoms and 1 Te atom; (b) 5 Se atoms and 1 S atom; and (c) 1 Se atom, 2 S atoms and 3 Te atoms. (d) The band structures of Cd doped high-entropy composition and the high-entropy composition without Cd. It is seen that Cd is more effective in widening the band gap and promoting the band convergence in $Pb_{26}CdSe_{13}Te_7S_7$-1 and $Pb_{26}CdSe_{13}Te_7S_7$-2 when it is bonded with several Se atoms. Considering the content of anion site, we adopt the result of $Pb_{26}CdSe_{13}Te_7S_7$-1 finally in the main text, and it is also the best SQS generated by ATAT.

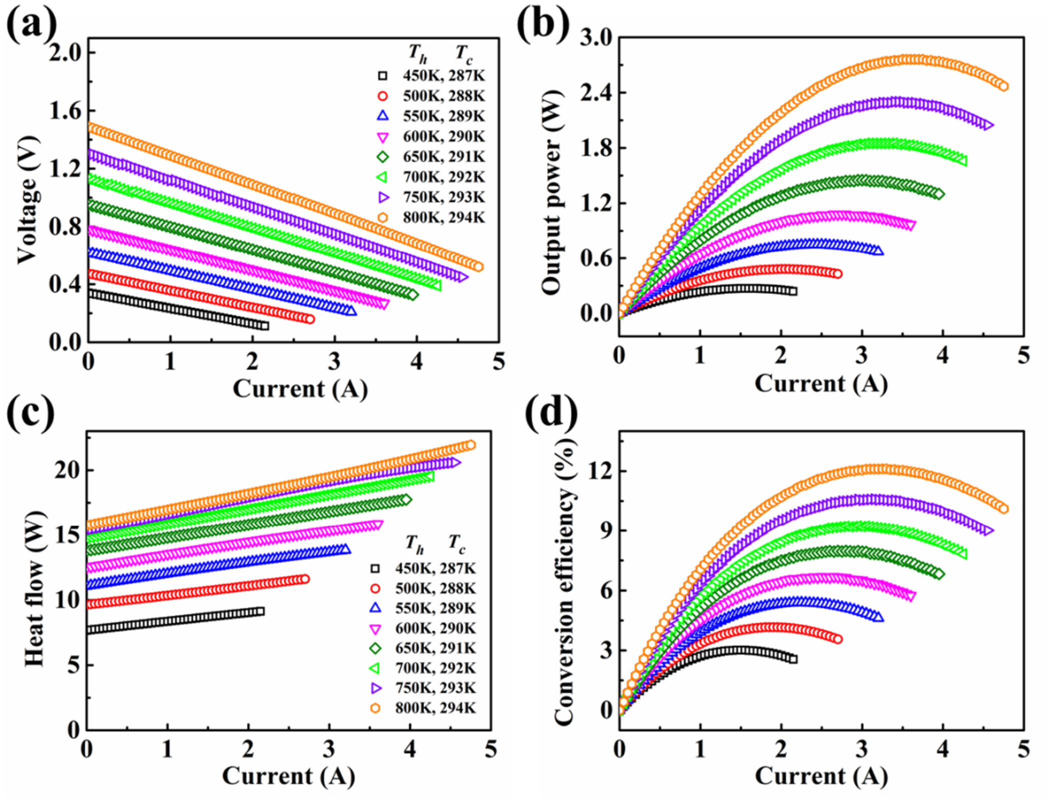

Supplementary Figure 3. Performance of the fabricated segmented thermoelectric module. Current dependence of (a) voltage, (b) output power, (c) heat flow at cold side, and (d) conversion efficiency.

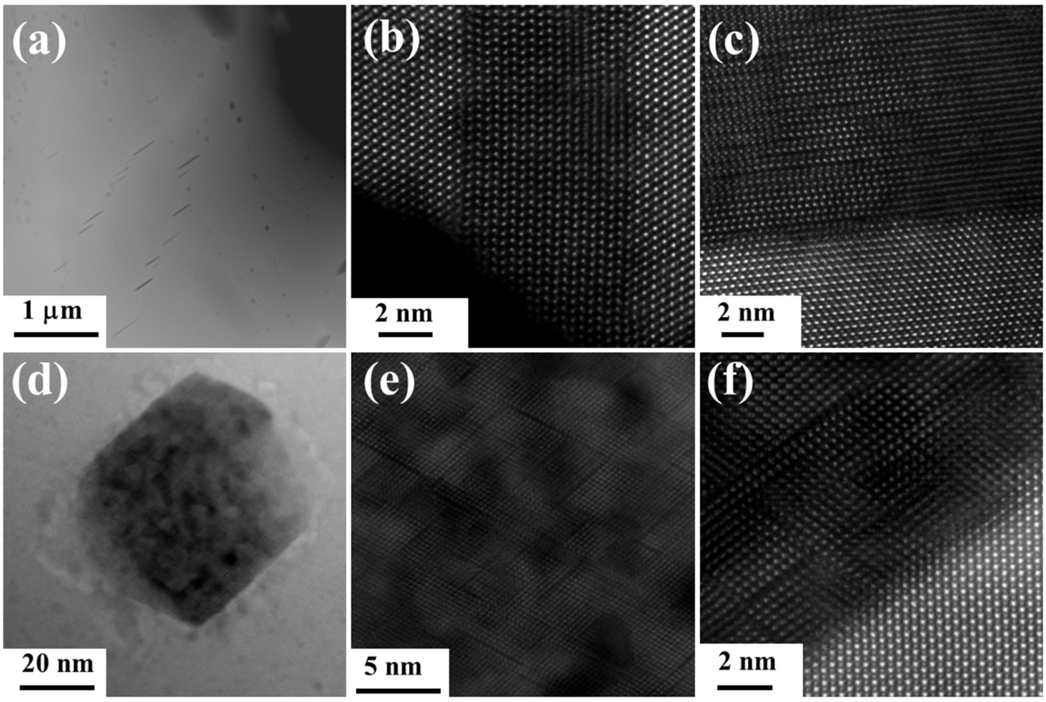

Supplementary Figure 4. Microstructure of $Pb_{0.935}Cd_{0.04}Na_{0.025}Se_{0.5}S_{0.25}Te_{0.25}$ sample. (a) Medium magnification STEM image which shows sphere-like and rod-like nanoprecipitates. (b) and (c) HAADF images of rod-like hexagonal nanoprecipitates without stacking faults. (d) HAADF image of a typical sphere-like nanoprecipitate. (e) The stacking faults within the sphere-like nanoprecipitates. (f) The semicoherent interface between sphere-like nanoprecipitates and high-entropy matrix.

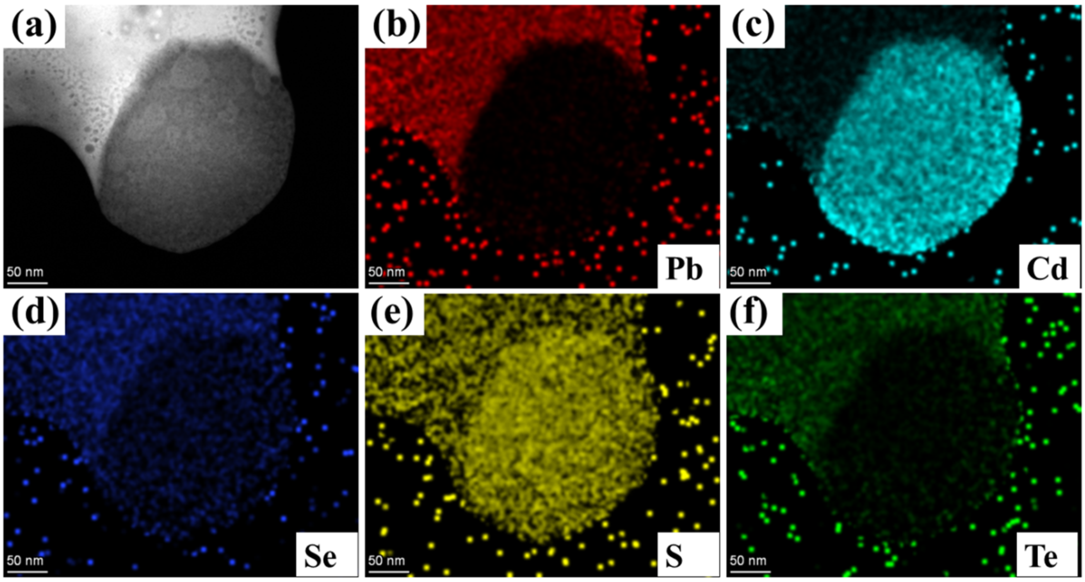

Supplementary Figure 5. EDS mapping of the sphere-like nanoprecipitate. (a) STEM image of a typical sphere-like nanoprecipitate. EDS mappings of (b) Pb, (c) Cd, (d) Se, (e) S, and (f) Te.

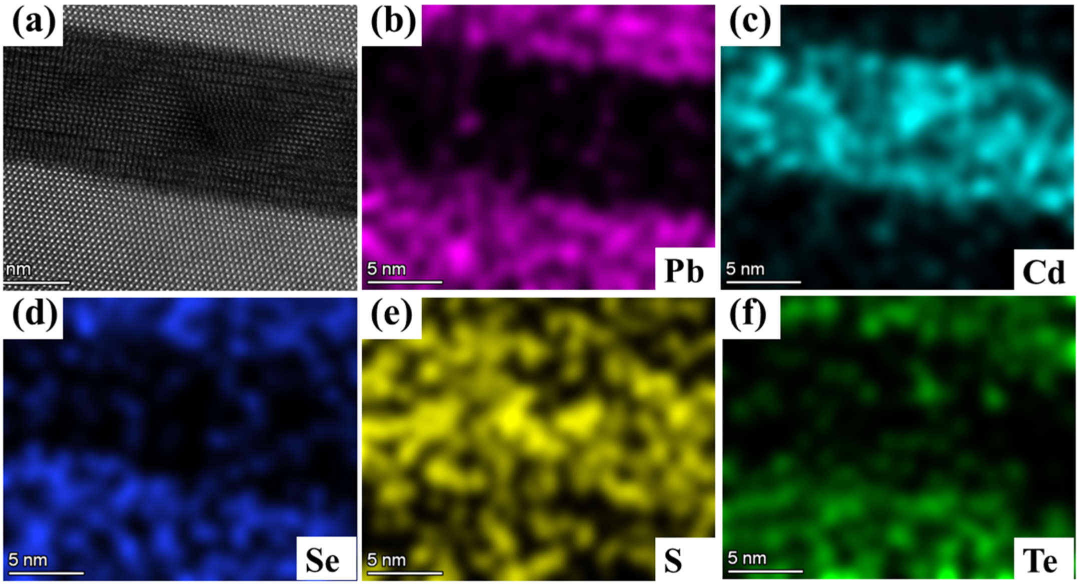

Supplementary Figure 6. EDS mapping of the rod-like nanoprecipitate. (a) STEM image of a typical rod-like nanoprecipitate. EDS mappings of (b) Pb, (c) Cd, (d) Se, (e) S, and (f) Te.

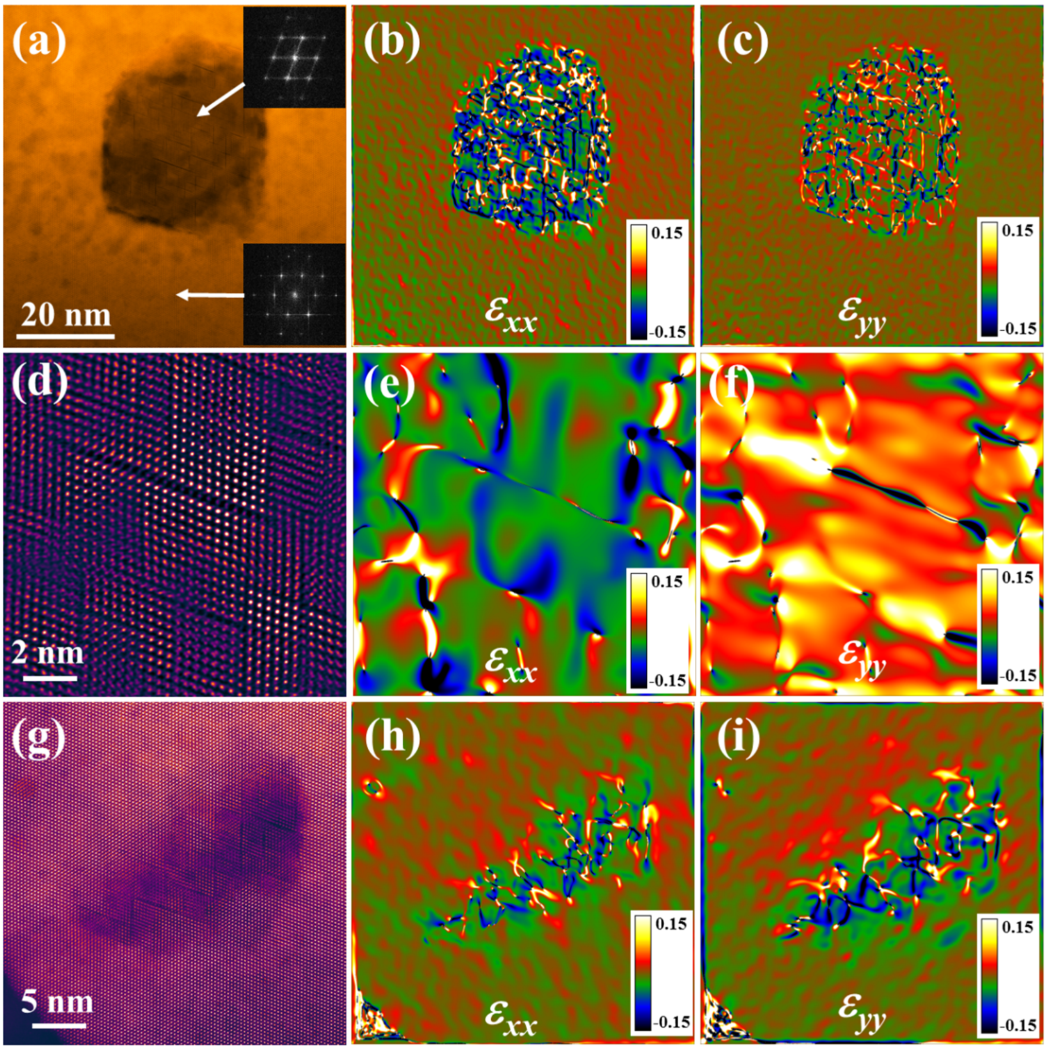

Supplementary Figure 7. HAADF images of (a) a large sphere-like nanoprecipitate, (d) stacking faults with the large sphere-like nanoprecipitate, and (g) a small sphere-like nanoprecipitate. The GPA results of (a) along (b) *xx* and (c) *yy* directions. The GPA results of (d) along (e) *xx* and (f) *yy* directions. The GPA results of (g) along (h) *xx* and (i) *yy* directions.

Supplementary References

1. Kresse, G., Furthmüller, J. Efficient iterative schemes for ab initio total-energy calculations using a plane-wave basis set. *Phys. Rev. B* **54**, 11169–11186 (1996).
2. Perdew, J. P., Burke, K., Ernzerhof, M. Generalized gradient approximation made simple. *Phys. Rev. Lett.* **77**, 3865–3868 (1996).
3. Monkhorst, H. J., Pack, J. D. Special points for Brillouin-zone integrations. *Phys. Rev. B* **13**, 5188–5192 (1976).
4. Zunger, A., Wei, S., Ferreira, L. G., Bernard, J. E. Special quasirandom structures. *Phys. Rev. Lett.* **65**, 353–356 (1990).
5. Ceperley, D. M., Alder, B. J. Ground state of the electron gas by a stochastic method. *Phys. Rev. Lett.* **45**, 566–569 (1980).
